# Interpersonal Motor Interactions Shape Multisensory Representations of the Peripersonal Space

**DOI:** 10.3390/brainsci11020255

**Published:** 2021-02-19

**Authors:** Martina Fanghella, Vanessa Era, Matteo Candidi

**Affiliations:** 1 Department of Psychology, Sapienza University, 00185 Rome, Italy; martina.fanghella@uniroma1.it (M.F.); vanessa.era90@gmail.com (V.E.); 2 IRCCS Fondazione Santa Lucia, 00179 Rome, Italy; 3 Department of Psychology, University of London, London EC1V 0HB, UK

**Keywords:** motor interactions, peripersonal space, multisensory integration, sensorimotor coordination

## Abstract

This perspective review focuses on the proposal that predictive multisensory integration occurring in one’s peripersonal space (PPS) supports individuals’ ability to efficiently interact with others, and that integrating sensorimotor signals from the interacting partners leads to the emergence of a shared representation of the PPS. To support this proposal, we first introduce the features of body and PPS representations that are relevant for interpersonal motor interactions. Then, we highlight the role of action planning and execution on the dynamic expansion of the PPS. We continue by presenting evidence of PPS modulations after tool use and review studies suggesting that PPS expansions may be accounted for by Bayesian sensory filtering through predictive coding. In the central section, we describe how this conceptual framework can be used to explain the mechanisms through which the PPS may be modulated by the actions of our interaction partner, in order to facilitate interpersonal coordination. Last, we discuss how this proposal may support recent evidence concerning PPS rigidity in Autism Spectrum Disorder (ASD) and its possible relationship with ASD individuals’ difficulties during interpersonal coordination. Future studies will need to clarify the mechanisms and neural underpinning of these dynamic, interpersonal modulations of the PPS.

## 1. Introduction

The present perspective review aims at proposing that interpersonal interactions may affect (sensorimotor) body and (multisensory) peripersonal space (PPS) representations. This perspective is grounded on two established piece of evidence regarding the plasticity of body and PPS representations, namely: (1) that synchronous visuo-tactile stimulation applied on ones’ own body and a fake (“rubber” or virtual) body part can induce changes in body representations, leading to the incorporation (i.e., feeling of ownership, proprioceptive drift, self-location drift) of the rubber body part in one’s own body representation (see Table 1); (2) that active tool use modulates the PPS by strengthening multisensory integration effects between visual [1,2] or auditory [3,4,5] stimuli delivered near the tool and tactile stimulation applied on the body, respectively, thus leading to the incorporation of the tool in the representation of the body of the user [2,6,7,8].

A relevant difference between the paradigms originally used to study the expansion of the PPS after tool use and the illusion of incorporating a rubber limb is that the first family of experiments implied the “active” use of a tool (however, see [4,9,10]) followed by measuring crossmodal congruency effects (CCE)] [11,12] (see Table 1 for a definition), while the second family of studies used “passive” visuo-tactile synchronous stimulations to induce subjective (ownership) or behavioural (proprioceptive drift, self-location drift) changes of body representation [13,14,15]. Regarding the role of different sensory modalities in modulating body representations, it has been shown that not only visuo-tactile, but also visuo-interoceptive synchrony may facilitate incorporation of external body parts. For example, seeing a virtual hand [16], body [17,18,19], or face [20] (but see [21]) pulsing in synchrony with participants’ heartbeat can increase their feeling of ownership towards it. As for the case of standard bodily illusions, the role of interoception (i.e., the inner sense of the physiological and visceral signals of the body, such as cardiac, respiratory, and gastric activity [22]) in triggering changes in body representations and body ownership has recently been addressed using immersive virtual reality set-ups. Monti and colleagues [23], for example, showed that breathing in synchrony with a virtual body (vs asynchrony) induces sense of ownership and agency of the virtual body, and that these effects depend on individuals’ interoceptive ability to perceive respiratory and cardiac signals. Conversely, the role of interoception in inducing the rubber hand illusion is debated [24]. It has been proposed that individuals with stronger interoceptive sensitivity are less likely to incorporate a rubber limb, suggesting stronger anchorage to own body in highly interoceptive individuals [25]. These results have been replicated during the enfacement illusion [26,27], and led to the proposal that interoceptive bodily signals may underlie self–other distinctions in the context of social interactions [28,29].

Here, we propose that interpersonal motor interactions entail temporarily exploiting the neural mechanisms underlying both multisensory integration involved in bodily illusions (i.e., the partner’s movement generates a corresponding sensation on my body, or inside my body) and tool incorporation (i.e., the partner’s movement and body are instrumental to achieve my goal as if the partner is a “tool” that I use). This would be translated in dynamic changes of own body and PPS representations, in order to facilitate sensorimotor predictions and accordingly adjust our behaviour to our partner’s movements [30]. In fact, in the context of interpersonal interactions finalised to shared goals, i.e., joint action [31], individuals’ sensorimotor channels need to become spatio-temporally aligned to facilitate interpersonal coordination. We suggest that acting together with a partner triggers plastic reorganisation of individual’s body and PPS boundaries, based on this spatio-temporal alignment, to form a joint representation of the agents’ bodies and the surrounding space. The proposed mechanism is in agreement with ‘we mode’ [32], or Shared Action Space (SAS) [33] models, proposing that interacting agents represent their individual actions together with aspects of the interactive scene, framing their movements, their representation of the space, and even their sense of the bodily self, in a co-constructed entity.

Consistently with this interpretation, and endorsing recent proposals supporting a Bayesian interpretation of body ownership [34], PPS [35,36], self [37,38], and action observation [39,40], we propose that temporary reorganisations of body and PPS representations through “predictive” multisensory integration of events occurring in one’s own action space support predictions of other people’s behaviour and mutual adjustments during motor interactions. Moreover, we suggest that this mechanism forms the basis for high-order mutual understanding, in particular the ability to infer the internal (i.e., motivational and intentional) causes of our partners’ behaviour. Importantly, Bayesian models of body ownership, PPS, and observed actions understanding are also useful to describe the mechanisms supporting self–other distinction, which are fundamental for efficient interpersonal interactions [41,42].

To support our proposal, we first briefly introduce the concept of peripersonal space, describe its neural basis, and summarise studies providing evidence for a tight link between the PPS and body representations (Section 2). In Section 3, we review the literature concerning changes in the PPS during action execution. Then, we link the notion that motor processes have a role in shaping body and PPS representations to evidence of plastic modifications of the body schema and the PPS after ‘incorporating’ tools through their active use (Section 4). Section 5 is devoted to describing the core idea of the present perspective review, i.e., that the pairing of motor and multisensory signals during interpersonal interactions may result in the emergence of a joint PPS. Here, we describe initial evidence of how interpersonal interactions trigger the formation of joint body and PPS representations. In Section 6, we further develop this idea by proposing that inter-individual sensorimotor interactions support higher-order forms of mutual understanding through the shaping of this shared PPS, based on multisensory integration [43,44,45,46]. This view is consistent with the idea that, during social interactions, understanding others’ internal states stems from a sensorimotor representation of their behaviour [47]. In the last section (Section 7), we link our proposal to the evidence that individuals with Autism Spectrum Disorder (ASD) show differences in PPS representations compared to neurotypical individuals [48,49,50].

**Table 1:** Keywords.

Concept	Definition
Rubber Hand Illusion (RHI)	A bodily illusion based on synchronous tactile stimulation of an unseen self-hand, and observed tactile stimulation of a rubber [13,14,15] or virtual [51] hand, placed in a congruent position with the real hand. This induces feeling of ownership of the fake hand and changes in where the real hand is perceived in the space (proprioceptive drift). Interestingly, the sense of ownership over a virtual hand in Virtual Reality (VR) can be induced by its mere observation in a first-person perspective, inducing visuo-proprioceptive congruency [52,53].
Rubber Foot Illusion	A bodily illusion based on the same multisensory integration principles of the RHI, but based on synchronous visuo-tactile stimulation or a rubber/virtual foot and consequent incorporation of the external foot [54,55,56].
Enfacement Illusion	An illusion based on multisensory integration of tactile stimuli felt on one’s own face and synchronous observation of tactile stimulation delivered on another face. This illusion induces incorporation of the partner’s face onto the person’s identity representation [57,58,59,60].
Full Body Illusion	A bodily illusion tested in immersive virtual reality, based on multisensory integration of tactile and visual information between stimulation received on the body and observed on a virtual body, inducing re-location of the self onto the virtual body, as shown by subjective (sense of ownership) and objective (self-location drift) measures [61,62,63,64,65]. Interestingly, the sense of ownership over a virtual body in VR can be induced by its mere observation in a first-person perspective, inducing visuo-proprioceptive congruency [66,67].
Embreathment Illusion	A bodily illusion based on synchronous or asynchronous breathing with a virtual avatar in immersive virtual reality, mediated by multisensory integration of interoceptive and visual cues, showing incorporation, ownership, and sense of agency of the virtual body after congruent respiration [23].
Crossmodal Congruency Effect (CCE)	The difference in reaction time in detecting a tactile stimulus on a body spot [11,12] when visual [2] or auditory stimuli [3,4,5,68] are presented on an incongruent location on the body.
Proprioceptive drift	A change of perceived hand location towards the rubber hand during the RHI [15].
Self-location drift	A shift of perceived full-body location towards the virtual body during the full-body illusion [64].

## 2. Body Representations and the PPS

Successfully interacting with the environment requires the online integration of sensorimotor information concerning one’s own body posture and position (i.e., the body schema) with the events happening and the objects located in the space around the body (PPS) [6,12,69,70]. The body schema has been defined as a specific type of body representation encoding the current posture of the body and its extension in space, based on the integration of somatic, proprioceptive, and tactile sensory information, implicated in guiding action [71,72,73,74]. Given the strong functional relation between the body schema and the representation of the space around the body upon which individuals can operate (PPS), the neural resources dedicated to representing the PPS and the body schema are functionally (and anatomically) interconnected (see [75,76] for reviews)

The notion of PPS is based on initial evidence of premotor and parietal bimodal visuo-tactile neurons responding to visual stimulation near the corresponding tactile receptive field in monkeys. These processes were later explored through multimodal (visuo-audio-tactile) integration of behavioural effects in (healthy and brain damaged) humans, and, more recently, by neuroimaging and brain stimulation studies in humans. Pioneering single-unit recording studies in monkeys described a class of multisensory, predominantly visual–tactile, neurons in premotor area 6 [77,78,79] parietal areas (Brodmann area 7b and the Ventral IntraParietal area (VIP)) [80,81] and the putamen [82]. These studies showed that the visual receptive fields of visuo-tactile premotor neurons were anchored to the tactile receptive fields (i.e., visual receptive field would respond to stimuli appearing near the part that was touched, no matter their retinal projection) [77]. These cells remain active even when the visual stimulus disappears [83] and respond to visual stimuli presented near a fake hand placed in front of the monkey in anatomical congruency with their own hand [84]. The majority of 7b visuo-tactile neurons are mostly active for stimuli approaching the face, arm, hand, and trunk, and this region contains bimodal neurons of which the visual fields might be either dependent or independent from the position of their tactile receptive fields [85]. The VIP contains a majority of bimodal neurons responding to stimuli near the head and face [86,87]. More recently, neuroimaging research on monkeys described an extended cortical network of occipital, parietal, premotor, and prefrontal areas, including somatosensory regions [88,89] (see [90] for a review), playing a key role in the definition of peripersonal space and supporting spatio-temporal predictions of the impact time of external objects [91].

In humans, behavioural and neuroimaging studies provided evidence of similar multisensory representations of the peripersonal space, which are mainly implemented in fronto-parietal cortical areas [92,93,94,95,96], reviewed in [76,97]. As in the case of monkeys, the human PPS is not a unique entity, and distinct neural systems selectively respond to events occurring in the space surrounding specific body parts [90]. Similarly to the segregation of different body parts observed in monkeys, studies suggest the existence of specific neural representations of the peri-trunk space [98], as well as the peri-face [99] and peri-hand [94,95,100], in humans, as confirmed by behavioural evidence [101]. Importantly, behavioural studies on the peri-hand space demonstrated that this region of space is sensitive to tool-use modulations [8,102].

In sum, since the PPS is organised in a body part-specific manner and it is affected by the position of the different body parts in space, body representations (i.e., the body schema) and the PPS are thought to be tightly linked functionally and underpinned by similar fronto-parietal networks. Nevertheless, important distinctions between body representations (with particular relation to the body schema) and the PPS have been recently reviewed [103].

## 3. The PPS Is Shaped by Action Planning and Execution

Relevant for the present perspective is the relation between the PPS and action execution. Specifically, extensive literature has described how the brain devotes resources to integrating multisensory processing with motor programmes to build unified representations of actions and the space in which these are performed (see [30,76,104] for reviews). For its role as a perception-to-action interface [105], the PPS has been recently proposed as an “action-value field”, i.e., a graded representation of the space according to event’s relevance for actions [104,106]. Coherently with this action-based model, representations of the PPS are sensitive to rapid recalibrations [107], reflecting the functional role of the peripersonal space as a spatial framework to create or avoid contact between objects and the body. Both avoidance and approach functions have been proposed for the PPS, based on the evidence that fronto-parietal regions responded during bodily protection behaviours [108,109] as well as during goal-directed action [70], leading to the proposal of a dual model of the peripersonal space (see [110] for a review).

For instance, it has been shown that, when an object enters the defensive peripersonal space (DPPS) around the face, it elicits a (subcortical) defensive response known as the hand-blink reflex [111,112]. This consists in an eye-blink elicited by the electrical stimulation of a hand, which is enhanced by proximity of the stimulated hand to the face. Interestingly, this automatic hand-blink reflex is triggered not only when the stimulated hand is near the self-face, but also to another person’s face, providing evidence for a shared substrate of self and others’ maps of PPS [112]. Importantly, this hand–blink reflex is modulated by the movement of the hand, so that the reflex is present when the hand which receives the tactile stimulation triggering the hand-blink reflex is moving toward the face, but it is absent when the hand occupies the same position but is moving away from the face [113].

On the other hand, clear online dependency of the dimension of the PPS on the planning and execution of actions was established by showing that planning and executing actions trigger a dynamic reorganisation of the peripersonal space [114,115,116,117,118,119]. Brozzoli and colleagues [115,116,119] employed a visuo-tactile integration task to measure the degree of interference caused by visual distractors placed over a to-be-grasped object on the detection of spatially congruent or incongruent tactile stimuli delivered on participants’ hands, during movement preparation and execution. These authors observed that the degree of interference between visual and tactile incongruent stimuli (CCE as defined in Table 1) was stronger during action planning and movement execution compared to when the object was merely shown to individuals. The authors interpreted these results as the consequence of a dynamic reorganisation of the peripersonal space around the far object, when the object becomes the target of ones’ own movement.

Remarkably, not only hand actions re-shape the PPS, but walking has also been shown to affect multisensory integration in the space surrounding the trunk [114]. Indeed, a study revealed that walking extended the behavioural effects of audio-tactile integration of stimuli perceived in the direction of locomotion, regardless of the coherence of the visual information [114]. Moreover, by combining reaching movements with walking toward the to-be-grasped object, Berger and colleagues [118] were able to show higher CCE at movement’s onset not only towards reaching targets, but also for walk-and-reach targets. However, these authors found that the PPS is not purely hand-centred with respect to orientation, such that when participants needed to change hand orientation to reach the object, the CCE decreased, and did not simply invert as expected.

All in all, the evidence shows that the PPS may be extended to external objects one is going to act upon, or the portion of space one is approaching. This literature bridges the role of action execution to the emergence of multisensory integration effects, normally occurring within the PPS, which were originally described after active tool use. We will review evidence concerning plastic reorganisations of the PPS after tool use in the next section.

## 4. Incorporating Tools in the PPS

In the previous section, we introduced the concepts of PPS as a dynamic multisensory representation of the space around the body, shaped by the actions of an individual. Before moving to the core of our perspective review, we now describe how body and PPS representations are sensitive to plastic reorganisations entailing the ‘incorporation’ of tools in ones’ own sensorimotor body representation. More in detail, we will suggest that the mechanisms supporting tool incorporation may share properties with those underlying the incorporation of the partner’s body parts when individuals actively interact.

Experimental evidence has shown that the body schema [120] and the peripersonal space [102] (for reviews see [76,97]) can be temporarily remapped during active or passive interaction [121] with a tool. This phenomenon was originally observed in monkeys [1] (for a review see [7]), and was later described in brain damaged [8,122] and healthy humans [2,5,120,123,124,125]. It has been suggested that modifications of the PPS underlying these effects depend on Hebbian plasticity [126,127,128], i.e., connectivity transformations driven by statistical associations of multisensory inputs from the environment. Plastic reorganisations of the PPS and the body schema after tool use are supported by evidence showing stronger CCE when a visual stimulus is presented near the used tool [8,122] or specifically next to the used part of the tool [10], while a tactile stimulus is delivered on the participants’ hand. Moreover, Cardinali and colleagues [120] showed that tool use not only changes the multisensory integration effects near the object (“extending” the PPS), but that it also affects motor indexes related to the body schema (i.e., action execution), and to the somatosensory body representation (i.e., increase of the represented length of the arm), once again supporting the close relation between the PPS and sensorimotor body representations. Interestingly, in a recent study, Miller and colleagues found that the somatosensory cortex responds to stimuli located beyond the physical body, and showed how, when a hand-held tool was touched, vibrotactile stimuli triggered activity in the primary and secondary somatosensory cortices of the participants [129]. Taken together, these results reveal how the body schema and the PPS can be temporarily extended in humans to incorporate external objects, which are useful needed to perform an action in the environment.

The similarities between the constructs of the PPS, the multisensory interface between the body and the space immediately surrounding it [97], and the body schema, a sensorimotor representation of the body finalised to action execution [6], and their sharing of anatomical and functional properties, made researchers even question hypothesize a dissociation of the two constructs [75]. Nevertheless, by studying tool use aftereffects or bodily illusions, several studies were able to partly disentangle the multisensory and sensorimotor representations underlying the PPS and different types of bodily representations. For instance, researchers aimed to understand whether the rubber hand illusion (based on multisensory integration) induces a change in the sensorimotor representations of the body (body schema). Kammers and colleagues [130] showed that the RHI does not affect reaching execution (based on the body schema), suggesting that visuo-tactile-proprioceptive illusions do not translate to alterations in body representations used to move (i.e., the RHI may affect the ‘body image’ but not the ‘body schema’ [73]). Conversely, a specific effect of tool use on the role of tactile information processing for motor control was studied by Cardinali and colleagues [131]. These authors showed that motor localisation of tactile stimuli on one’s limb is affected after tool use as if the limb has extended, and that this effect is not observed in case the localisation follows a verbal indication. By studying a deafferented patient, these authors have also shown that processing proprioceptive information is a necessary condition to support body schema plastic changes after tool use [132].

Similarly, pairing of motor efferent signals with perceived sensory consequences may also affect the rubber hand illusion, such as in the moving and virtual Rubber Hand illusions [51,133,134,135,136,137,138]. The relation between the role of efferent motor and afferent sensory signals to the incorporation process has been recently cast in the perspective of Bayesian sensory filtering through predictive coding [139]. This study showed that task-relevant distal cues could affect the sense of body ownership of a virtual hand during action execution, providing evidence that forward models of body ownership are formed not only through integration of internal (motor) and proximal (tactile and proprioceptive) cues, but also of distal (visual and auditory) ones, if they are informative of the action’s outcome [139].

This evidence reveals how the brain integrates sensory information from external and internal bodily cues during action execution, suggesting that generative models of body ownership are updated through upcoming information from multiple proximal (proprioceptive, tactile) and distal (visual, auditory) channels, when the agent pursuits goal-oriented actions in the environment. Thus, we note that while synchronous visuo-tactile stimulations might be sufficient per se (see [121]) to extend the PPS over an object, it is likely that concurrent visual, tactile, proprioceptive, auditory, and motor information, paired in time during natural (transitive) tool use, underlie the incorporation of the tool in a user’s PPS.

Given the action-based [104,114,115,116,117,118] and predictive nature of the PPS [90,109], researchers have proposed the Bayesian framework as a useful model to interpret the emergence and structure of the PPS [35,140,141], as in the case of self and body representations [34,37,38,142]. Specifically, according to these proposals, the PPS would act as a multisensory coupling prior, sensitive to recalibrations driven by experience [107,126,127]. Coherently with a predictive view of the PPS, it has been shown that forward models extend predictive mechanisms of multisensory integration, related to the body, to hand-held tools, such that self-touch reduces sensory perception either when performed with the hand or an incorporated tool [143]. This is explained by the fact that Bayesian priors inherently adapt to a dynamic environment in which sensory expectations are updated though experience, linking multisensory perception to action.

Expanding the idea of the PPS as a space where events may enter in contact with ones’ body [109] this evidence supports the idea that motor capabilities shape the way the brain dedicates multimodal processing of events that will enter in contact with one’s body. This makes the PPS the perfect candidate to support our ability to coordinate our behaviour with our interaction partner’s actions. In fact, during interpersonal interactions, we need to predict which when and where the movements of our partner will generate events that we will feel on our body, whether we are directly touching the body of our partner, or whether we are using an object to interact. Consistently, predictive accounts of interpersonal interactions have already been proposed for these scenarios [43,144].

The tight functional link between visuo-audio-tactile and proprioceptive integration and motor control leads to the idea that extensions of the body schema and the PPS (such as after active tool use) may contribute to support our ability to interact with others. The next section is dedicated to building the proposal that the multisensory integration underlying tool incorporation may be extended to the incorporation of a partner body part during interpersonal interactions. Indeed, during interpersonal interactions, visuo-tactile and auditory events are perceived as a function of one’s own, but also other people’s actions. In these cases, one’s own sensory inflow co-occurs with the movements of a partner, thus establishing statistical associations between one’s own motor command and the effects of the behaviour of a partner.

## 5. The PPS Is Modulated by Motor Interactions

Several researchers have suggested that our body and other people’s bodies, and the space surrounding them, may be represented in a common framework [30,145]. According to Gallagher, the idea of joint body representations has its philosophical roots in Merleau-Ponty’s notion of intercorporeity [146]. Merleau-Ponty considers intercorporeity to be a pre-reflective, relational phenomenon, which he defines as “an internal relation that makes the other person appear as the completion of the system” ([147], p. 368). This concept is now relevant for cognitive neuroscientists interested in disclosing the neuro-cognitive mechanisms underlying social interactions.

Thomas, Press, and Haggard investigated the existence of a shared representation of our own body and the other’s body (and PPS), by investigating the degree of visuo-tactile integration between tactile stimuli delivered on the participants’ body and observed visual stimuli appearing near a facing model’s body [148]. Participants exhibited faster responses to tactile events on their own body after a visual event that was presented in the corresponding anatomical (e.g., left hand–left hand) location on the model’s body, compared to a non-corresponding location. This shared representation of the body was expanded by a study investigating whether shared sensory experiences between two people, induced by the enfacement illusion, could trigger the remapping of one’s own peripersonal space around the other’s body [149]. Crucially, results showed an increase in audio-tactile integration in the space close to the confederate’s body after the shared experience, demonstrating a temporary remapping of one’s own PPS around the confederate’s body. Coherently with these behavioural effects, an fMRI study identified shared pattern of activity in the left ventral premotor cortex for processing events occurring in self and other people’s peripersonal space, but also activations in the anterior cingulate cortex specific for processing information related to the space surrounding other people’s bodies [145].

Interestingly, the impact of sensory sharing on these shared body and PPS representations is modulated by higher-order factors such as the social context. In a series of experiments, by using an audio-tactile integration task, Teneggi and colleagues [150] described how individuals’ peripersonal space is differently modulated by the presence of another individual or a mannequin. Then, they showed that the boundaries between self and the other’s peripersonal spaces merged after playing an economic game with another person, but only if this person behaved cooperatively. These observations were corroborated by another study, showing that modulations of the peripersonal space in presence of others was modulated by people’s perceived morality [151]. These results revealed that PPS representations are sensitive to top-down modulations related to social information, showing a link between low-level multisensory processing and high-level social cognition.

Shared multimodal body and space representations may be extremely relevant to support interpersonal interactions, where the movements and sensory events happening on a partner’s body need to be integrated with one’s own actions. Indeed, interacting successfully with others requires to predict the outcomes of other people’s actions in order to facilitate mutual adjustments and motor coordination between partners [152,153]. Previous studies showed how sensorimotor resonance may support action prediction not only in the context of action observation [39] but also during joint action, in order to facilitate interpersonal coordination [31]. According to this hypothesis, individuals engaging in joint action would manage to understand and predict the actions of their partners and consequently adjust their behaviour by representing aspects of the interactive scene in a ‘we-mode’ [32]. As Pezzulo and colleagues [33] suggested, social interactions are embedded in a shared representation of the space, the ‘Shared Action Spaces’ (SAS), which supports crucial computations underlying interpersonal interactions. Specifically, they propose that, during joint actions, the mechanisms for sensorimotor transformations and multisensory integrations incorporate information relative to the co-actors and induce a recalibration of individual spatial representations onto a shared one, re-referenced on the dyad. Crucially, sensorimotor transformations in the SAS enable real-time coordination, because they enclose predictions about the partner’s future actions.

**Figure 1:**
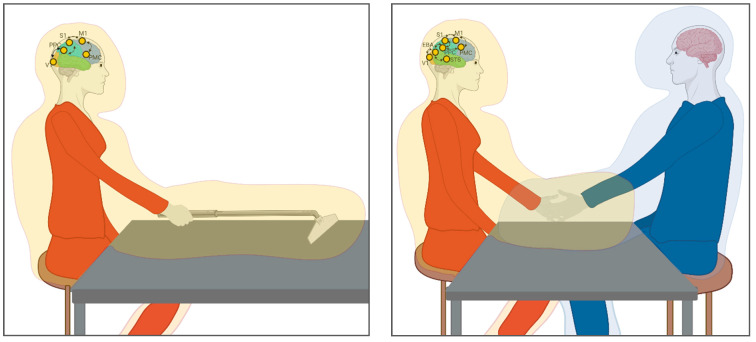
Plastic changes in the peripersonal space (PPS) during tool-use and interpersonal motor interactions. **Left panel**: Incorporation of a tool in own PPS during active tool use. **Right panel**: The same mechanism could underlie the incorporation of the partners’ limb during motor interactions (i.e., handshake), to facilitate interpersonal coordination. The neural network involves feedforward and feedback projections between visual (V1, Extrastriate Body Area (EBA), Superior Temporal Sulcus (STS)), somatosensory (S1), and motor (M1) areas, as well as multisensory areas involved in representing the PPS, i.e., the posterior parietal cortex (PPC) and premotor cortex (PMC). The orange/blue shadow represents the PPS expanded around the tool or partner (Created with Biorender.com).

In line with these accounts, we propose that plastic reorganisations of the body schema and the surrounding peripersonal space support interpersonal attunement between partners in the context of joint actions. This phenomenon would be supported by temporary reorganisations of individual bodily and spatial maps onto a shared representation, having implications beyond plastic reorganisation of the body and PPS shaped by ‘tool use’ (i.e., incorporating the partner’s body in a shared self-other representation). For a graphical dep iction, see Figure 1.

In the context of the emerging field of ‘two persons neuroscience’ [154,155,156], interactive paradigms have been developed to investigate the neurocognitive mechanisms supporting dyadic or group interactions and these paradigms have been also used to study changes in body and PPS representations in social contexts. The first study that tested the effect of the presence of another person inside (or outside) participants’ PPS on individuals’ visuo-tactile CCE showed that multisensory integration is reduced while performing a tactile detection task when another individual was performing a complementary task within the participant’s PPS [157]. More recently, evidence of the formation of a shared PPS (or an ‘entangled’ body schema, as the authors call it) after interpersonal interactions comes from a behavioural study, where two individuals had to synchronise their pulling of a rope to cut a candle (i.e., interpersonal visuo-motor and proprioceptive coupling) and were then tested for incongruent visuo-tactile stimulation effects on their partner’s hand [158]. Interestingly, the interaction increased the interference effect of incongruent visual stimuli occurring near the partners’ (used for the interaction) index or thumb finger on the detection of tactile stimuli on the participant’s (unused for the interaction) thumb or index finger indicating that individuals remapped the space around the partners’ body on their corresponding body part. This bodily entanglement was found especially in participants with high interdependent self-construal levels, suggesting that lower-level multisensory bounding is modulated by higher-order social representations of the self. To further investigate the effects of interpersonal sensorimotor sharing on individuals’ body schema, in two other experiments, Soliman and colleagues [158] measured the degree of visuo-motor interference (that is, the automatic simulation of other’s movements during action execution) [159] after the joint and the solo sawing conditions, asking their participants to execute a movement while observing the partner performing a different one. Participants’ performance was more affected by the observation of an incongruent action after the joint sawing task compared to the solo control condition, suggesting that the interaction affected individuals’ motor representations of the partner’s movements. These results are in line with previous studies showing that visuo-motor interference effects automatically arise when participants coordinate their actions with a partner to perform complementary (incongruent) interactions [160,161,162,163,164,165,166,167,168,169,170]. Interestingly, these effects are present only in situations requiring predictions of the partner’s action [163,167], highlighting the link between motor simulation and action prediction during motor interactions [171,172,173].

The results from Soliman and colleagues suggest that the effects of interpersonal coordination on plastic reorganisations of the body schema persist over the completion of the task. It is not clear whether this ‘entanglement’ effect [158] (i.e., the persistence of a shared representation of the body schema beyond the completion of the interactive task) may facilitate long-lasting interpersonal coordination and social bonding [174]. Recent research showed that long-term experience with a tool stably modifies peripersonal space [124] and modulates internal models of sensorimotor representations, which drive the tool-based action [125]. In interpersonal interactions, the long-lasting effects of interpersonal entrainment [175] on social behaviour have been discussed in a recent review [176], suggesting its role in promoting social bonding and prosocial behaviour.

Recently, CCE modulations have been used in the context of interpersonal paradigms that involve grasping, or observing a partner grasping, an object to study the role of the object’s ownership in modulating individuals’ PPS representation. Extending previous studies on the role of reaching actions in shaping PPS [115,116,117], researchers have developed a paradigm where individuals act in turn with a partner on an object [177]. They assessed changes in the PPS through a visuo-tactile task, while dyads of participants either grasped or observed a partner grasping an object, whose ownership was experimentally assigned to either one of the two partners (individual ownership), or to both partners (shared ownership). Interestingly, when ownership was assigned exclusively to one participant, a stronger CCE emerged when grasping one’s own object and observing others grasping their own object. Instead, no modulations of CCE were found when grasping and observing to grasp an object that was not one’s own. However, when ownership was equally assigned to the two participants, the CCE modulation emerged both when the action toward the shared object was executed or observed.

Another study has shown that the PPS is also sensitive to more abstract forms of interactions, such as music making. Participants showed plastic reorganisations of the PPS (i.e., audio-tactile congruency effect) after joint jazz performance [178]. More specifically, the authors asked dyads of musicians to perform a jazz improvisation in a cooperative (correct harmony) or uncooperative (incorrect harmony) condition and tested plastic reorganisation of the peripersonal space through an audio-tactile integration task, by measuring reaction times to tactile stimuli on the subjects’ right hand and auditory stimuli delivered at two different distances, next to the subject and next to the partner. Results showed an increase of reaction times to tactile-auditory stimuli presented near the partners’ body (indexing poor crossmodal integration) only following the uncooperative condition. The authors interpreted this result as a multisensory marker of withdrawal from an uncooperative partner.

These studies indicate that crossmodal integration mechanisms characterising the representation of the space surrounding our body may be modulated by sensory sharing and sensorimotor coupling during interactions with others, suggesting that the emergence of interpersonal multisensory integration processes may support fundamental action predictions during joint actions. In this respect, we speculate that the left anterior intraparietal sulcus (aIPS), known to code for the goal of executed [179,180] and observed actions [181], as well as to support the ability to perform interpersonal complementary motor interactions [162,167,182,183] might be involved in integrating sensorimotor information of observed and executed actions. Crucially, the studies mentioned above highlighted a causal contribution of this region in supporting complementary motor interactions where individuals need to mediate incongruent visuo-motor information.

Other studies have addressed the neural underpinnings of interpersonal coordination during the occurrence of unpredicted events, which represent another situation where sensory events need to be integrated with ones’ own movements. Indeed, when interacting with others, we sometimes need to deal with their errors, which represent discrepancies between expected and executed actions. Thus, motor interactions necessarily require predicting and monitoring the interactor’s actions. Electroencephalographic (EEG) studies highlighted the presence of specific fronto-central markers (in particular theta/alpha synchronisation) occurring when performing errors [184] or observing errors performed by one’s own avatar in a first-person perspective [185,186,187,188]. Furthermore, recent studies using transcranial alternating current stimulation provided evidence for a causal role of midfrontal theta activity during conflict monitoring [189], and a causal role of midfrontal and occipito-temporal theta activity (observed when participants are presented with stimuli depicting hands in EEG studies, [190,191]) in a task where conflict is elicited by hand stimuli [192].

Importantly for the present review, a recent study showed that the same fronto-central electroencephalographic markers (in particular theta/alpha synchronisation) registered when performing or observing errors also emerge during motor interactions [193], when prediction and monitoring of the partner’s action is needed [194]. It is relevant to note that the source of the registered theta/alpha activity included frontal and occipito-temporal regions (i.e., the Extrastiate Body Area) [193], suggesting their putative role in integrating visual and motor information during motor interactions [195]. Taken together, these studies highlight a fundamental role of brain regions implicated in visuo-motor transformation, known to be linked to multisensory body and PPS representations (see [97]), in supporting the ability to efficiently interact with others.

In this respect, Dumas and colleagues [196] recently provided empirical evidence for a unified model of sensorimotor and high-order cognitive processes underlying interpersonal coordination. The authors investigated the behavioural and neural mechanisms underlying a human–avatar interaction, and found a link between sensorimotor representations and attribution of intentions at a behavioural and at a neural level. Specifically, behavioural results highlighted a correlation between sensorimotor performance and the correct attribution of intention, and whole-scalp connectivity analysis of EEG data highlighted that large-scale connectivity modulations were associated with both top-down (social cognition) and bottom-up (sensorimotor) aspects during live interactions.

In the next section, we will describe how unified models of sensorimotor/multisensory and high-order cognitive processes are well-captured by the predictive coding framework [197,198], which may also account for the core mechanisms underlying interpersonal motor interactions [199,200].

## 6. Predictive Coding Accounts of PPS and Their Possible Role for Interpersonal Interactions

Predictive coding is a computational framework that allows to explain sensorimotor processes as the brain attempts to minimise prediction errors through the generation of internal representations of the hidden causes of sensory inflow, i.e., priors [201]. When applied to the case of interpersonal interactions, it is plausible that the success of interactions rests on the accuracy of our models of the causes of other people’s behaviour. Specifically, in this context, priors are conceivable as models of the internal (motivational, emotional, cognitive) causes of other people’s behaviour, which are coupled to our sensory inflow through forward and backward connections [39,40,201,202]. Predictive coding is a framework based on minimising prediction error though recurrent interactions among different levels of a neuronal hierarchical architecture. Specifically, each level of this hierarchy employs a generative model to produce a prediction in the lower level. The generative model is connected to the lower level through backward connections, in order to compare the higher-order prediction to the lower-level representation and generate a prediction error. This prediction error is then sent to the higher level, via forward connections, to adjust the neuronal representation of the causes of sensory information (prior). This reciprocal exchange of signals continues until the prediction error is minimised and the most likely cause of the input has been modelled [39,201,203].

Originally, predictive coding accounts of the mirror neuron system (MNS) provided a hierarchical and unified neurocognitive architecture of sensorimotor transformations (involving the STS, premotor, and parietal areas) to recognise other people’s intentions and action’s goals [39,40]. As the somatosensory system is now considered part of this simulative network [204,205], Ishida and colleagues proposed a predictive coding account of shared body representations that include parietal and insular regions, integrating exteroceptive, proprioceptive, and interoceptive information to create shared, or distinct, bodily and affective representations [41]. Importantly, it has been proposed that interpersonal predictive coding may underlie interpersonal synchronisation between the interactive partners. This would be achieved through a mutual exchange of sensory signals, generating reciprocal predictions on the partner’s behaviour, which is reflected in interpersonal synchrony between neuronal states [199].

More recently, the PPS has been conceptualised, and tested, as a prior for coupling visual and proprioceptive systems, allowing for the computation of the probability that visual and proprioceptive signals are associated with each other [140]. Through statistical learning of paired visual and proprioceptive information during action execution, this prior would be updated from incoming sensory information during adaptive PPS recalibrations [35,107], including PPS expansions to external objects.

Indeed, in the context of interpersonal motor interactions, the brain is challenged with the need to integrate visual or auditory (i.e., distal) and tactile-proprioceptive (i.e., proximal) information from the two partners, while behaving to achieve goals. In case these events are synchronised in time and repeated over time, the brain may solve this challenge by generating a joint representation of the two agents’ PPS, forming a prior, which enables predictions on the incoming sensory information generated not only from self-actions, but also from the partner’s actions, within a unified model. This idea is reminiscent of the fact that sensory sharing may enlarge PPS [149] and that humans seem to code other people’s PPS too [145].

One way to realise sensorimotor sharing is to reuse one’s own internal representations of “what it is like” to perform the action of our interactive partner (i.e., motor and somatic simulation). A recent study adopting an interactive paradigm explored how individual or shared predictive models enable compensatory movements while two partners lift a glass-like object from the partner’s tray, either simultaneously or sequentially, or from their own tray [206]. Results showed that participants’ compensatory movements to balance the tray while the partner was lifting the glass were reduced when they were simultaneously lifting the partners’ glass, compared to lifting each other’s glasses sequentially. This result indicates that performing the action allowed participants to access sensorimotor information paired with the movement, which was used to predict and accordingly adjust for the effects of the other person’s lifting. Moreover, the authors interpreted these results as evidence to support the hypothesis that co-actors did not combine two sets of forward models (one for self-movement and one for the other’s movement) to predict the outcome of the joint action, but reused a bimanual model (i.e., a model which generates predictions on the outcome of their movement when they lifted the glass from their own tray) while performing simultaneous joint action, thus using a unified model to generate predictions on the synchronous lifting. Conversely, in the sequential condition, when lifting and balancing were performed in turns, the participants used two separate, unimanual models. The authors suggested that the bimanual model was more effective in making predictions on the outcome of the partners’ lifting, compared to the unimanual model, because it formed a joint motor plan, where sensory information from self-movement was used to make predictions on the partners’ movement. Crucially, this experiment provided the first evidence for an ‘agent neutral’ predictive model of joint action.

Interpersonal predictive coding could also play a role in the development of social skills during infancy. Recent computational views of typical and atypical predictive learning [45,207] propose that higher-order social functions develop from becoming able to master the laws of interpersonal sensorimotor coupling. Specifically, the authors suggested that predictive learning of sensorimotor signals plays a key role in early cognitive development, in particular in distinguishing the self from others, imitating gestures, understanding other people’s actions, and sharing emotions. Importantly, these skills and the underlying neural systems develop through primary social interactions, which are grounded in sensorimotor mutual exchanges between the infant and caregiver [208,209].

## 7. Plastic Representations of the Body and PPS in Typical and Atypical Development

In the previous sections, we proposed that interpersonal motor interactions induce plastic reorganisations of body and PPS representations, and, on the basis of predictive coding accounts, we outlined how high-order social communication may be grounded in low-level, interpersonal embodied processing. Here, we aim at exploring how these processes may operate differently in individuals with atypical neurodevelopment (i.e., Autism Spectrum Disorder). Autism Spectrum Disorder (ASD) is a neurodevelopmental condition characterised by difficulties in social interaction and communication, as well as restricted interests and repetitive behaviours [210]. Differences in how individuals with autism process sensory and social information have been extensively shown, for instance in the domain of touch [211], vision [212], auditory stimuli [213], as well as social stimuli, including faces [214,215], gaze [216,217], biological motion [218,219], emotional body language [220,221], and speech prosody [222].

Relevant for this work is that individuals with Autism Spectrum Disorder show reduced bodily illusions [223,224] and smaller and more sharply defined PPS [48,49,223]. In a recent experiment, Mul and colleagues [223] investigated autistic individuals’ sensitivity to the full bodily illusion (FBI). Participants with ASD showed to be less susceptible to the FBI, as highlighted by lower scores in questionnaires of self-identification with the virtual body, and reduced changes in self-location. Moreover, ASD participants were also characterised by a smaller PPS. Interestingly, the degree of identification with the virtual body was positively correlated with individuals’ empathic traits. The authors interpreted the observed reduced plasticity of body and PPS boundaries as a marker of more pronounced self–other distinction. These results partly replicate previous findings showing delayed plastic modulations of body representations after the rubber hand illusion in children with ASD compared to Typically Developing (TD) children, and a significant association between reduced susceptibility to the RHI and lower empathy [224]. Importantly, it has been shown that atypical multisensory integration in ASD encompasses interoceptive signals [225], having implications for empathy as well [226].

These differences in body and PPS plasticity may have important implication for difficulties in developing interpersonal motor coordination skills characterising ASD. For instance, Curioni and colleauges [227] tested pairs of individuals with and without autism in a social coordination task, where participants engaged in a joint grasping task, where each participant was either in charge of performing a movement in accord with a received instruction (coordinating in time) or adapting to the partner’s movement (coordinating in time and space). The results of this study highlighted that the strength of autistic traits negatively correlates with participants’ ability to modulate their behaviour according to their role in the interaction, suggesting reduced disposition to attune with the partner in individuals with stronger autistic traits.

Initial evidence of the lack of plastic modulations of the PPS in a social context in autistic individuals is provided by a recent work by Noel and colleagues [50], who used EEG to investigate changes in the PPS induced by the presence of another individual in two groups of ASD and TD. Participants engaged in a tactile detection task while visual stimuli were presented in the near and far space in a social (the experimenter sat in front of them, at a distance of 150 cm) or non-social situation (they performed the task being alone in the room). In line with their hypothesis, electrophysiological markers of PPS remapping, reflecting changes in neural activity underlying multisensory processing, were modulated by the social context in TD but not ASD individuals, confirming inflexibility of their PPS in the presence of others. Moreover, the authors proposed a biologically plausible neural network of the observed EEG responses, based on Hebbian plasticity, highlighting that the PPS rigidity in ASD would be based on changes in excitatory and inhibitory connections at the level of multimodal areas. More broadly, the authors interpret their findings in the framework of a Bayesian account and suggest an inflexible updating of priors in ASD.

Remarkably, a Bayesian account of autism have been previously proposed [228,229]. Pellicano and Burr [228] first suggested that atypical sensory processing in autism might be explained in terms of weaker (hypo) Bayesian priors, i.e., when processing current sensory information, autistic individuals rely less on internal models based on previous sensory experience. This hypothesis provides an appropriate explanation for hypersensitivity to sensory information characterising autism [230,231,232,233] having implications for social difficulties as well [230,234], although hyposensitivity to sensory stimuli has also been observed [235], see [236] for a review. This model was then reformulated within the predictive coding framework [237,238,239,240], providing a unified account of atypical sensory, cognitive, and social computations in ASD [241].

The Bayesian account of intersubjectivity recently proposed by Bolis and Schilbach [241,242] is of particular interest for the scope of this work. In particular, the authors propose that poor interpersonal coupling in social interactions in ASD compared to TD might be a result of different predictive styles across these populations. This proposal is grounded in the idea that social interactions are a key factor in the formation of consciousness and higher-order human psychological processes [243]. This idea also has many antecedents in the domain of attachment during infant development, such as Bowlby’s attachment theory [244,245,246], arguing that early interactions with the caregiver shape our cognitive and affective style in social interactions during further stages of development.

More recently, Fotopoulou and Tsakiris [42] proposed that embodied interactions with other people in early infancy shape our capacity to distinguish the self and other, and contribute to building the bodily sense of self. In their account, feelings of body ownership may develop through early multisensory integration mechanisms, encompassing exteroceptive (i.e., visual, auditory), proprioceptive, and interoceptive signals [42]. In accord with this idea, multisensory integration mechanism in infants have been described in a study showing that infants look preferentially at visual face stimuli being touched in synchrony with their own face and are able to discriminate visuo-tactile synchrony from visual-tactile asynchrony [247]. According to Fotopoulou and Tsakiris’ account, during parents–child interactions in early infancy, caregivers offer naturalistic “matching” between multisensory stimuli in an interactive frame, such as the experience of perceiving tickling and giggling at the same time. These experiences would underlie early mentalisation of one’s own body, and differentiation with other people’s bodies, structured as a Bayesian inference enabling self or other attribution of the sensory experience through statistical learning.

With relation to ASD, it has been proposed that early interactions between autistic toddlers and the social environment would be characterised by reduced innate orientation towards social stimuli [248,249] during early stages of development, having cascade effects on the maturation of the so-called social brain. Specifically, this reduced engagement with the social environment may shape differently ASD sensory and social processing [250]. Future research should investigate the role of early interpersonal sensorimotor interactions in shaping the mechanism of the underlying body and PPS representations in the typical population and neurodevelopmental and psychiatric conditions.

## 8. Conclusions

The main purpose of this work was to develop a conceptual framework for the hypothesis of a joint PPS, which would arise to facilitate interpersonal coordination during joint action. In particular, we proposed that this mechanism would have advantages for predicting and aligning with the partners’ actions, with potential implications for inferring their high-order mental states. First, we described how multisensory representations of the body and PPS are modulated by actions, and showed how the body schema and the peripersonal space can be temporarily extended to incorporate tools, or other body parts, for instance during bodily illusions. We proposed the hypothesis of the formation of shared body and peripersonal space representations during interpersonal interaction and discussed findings suggesting the creation of an ‘entangled’ body schema and joint PPS, after two individuals engaged in a task which required interpersonal sensorimotor coordination. Then, we reviewed how body and PPS representations have been interpreted in the context of predictive coding frameworks, which may have important implications for mutual coordination during interpersonal interactions. Finally, we considered how this mechanism might operate differently in individuals with autism spectrum disorder, with relation to traditional and contemporary theories emphasising the role of early interactions in constructing embodied representations of self and others.

Future research will need to expand our knowledge of the neurophysiological, behavioural and computational mechanisms underlying plastic reorganisation of the PPS during and after joint action, linking multisensory and sensorimotor representations to feelings, intentions, and other high-order mental states, in typical and atypical development.
